# Pre- and post-surgery brain tumor multimodal magnetic resonance imaging data optimized for large scale computational modelling

**DOI:** 10.1038/s41597-022-01806-4

**Published:** 2022-11-05

**Authors:** Hannelore Aerts, Nigel Colenbier, Hannes Almgren, Thijs Dhollander, Javier Rasero Daparte, Kenzo Clauw, Amogh Johri, Jil Meier, Jessica Palmer, Michael Schirner, Petra Ritter, Daniele Marinazzo

**Affiliations:** 1 Department of Data Analysis, Ghent University, Ghent, Belgium; 2 Research Center for Motor Control and Neuroplasticity, KU Leuven, Leuven, Belgium; 3 IRCSS San Camillo Hospital, Venice, Italy; 4 Department of Clinical Neurosciences, University of Calgary, Calgary, Alberta Canada; 5 Hothckiss Brain Institute, Cumming School of Medicine, University of Calgary, Calgary, Alberta Canada; 6 Murdoch Children’s Research Institute | MCRI Research Group for Developmental Imaging, Melbourne, Australia; 7 CoAx Lab, Carnegie Mellon University, Pittsburgh, USA; 8 Indraprastha Institute of Information Technology, Delhi, India; 9 Berlin Institute of Health at Charité – Universitätsmedizin Berlin, Charitéplatz 1, 10117 Berlin, Germany; 10 Charité – Universitätsmedizin Berlin, corporate member of Freie Universität Berlin and Humboldt Universität zu Berlin, Department of Neurology with Experimental Neurology, Charitéplatz 1, 10117 Berlin, Germany; 11 Bernstein Center for Computational Neuroscience, Berlin, Germany; 12 Einstein Center for Neuroscience Berlin, Charitéplatz 1, 10117 Berlin, Germany; 13 Einstein Center Digital Future, Wilhelmstraße 67, 10117 Berlin, Germany

**Keywords:** CNS cancer, Network models

## Abstract

We present a dataset of magnetic resonance imaging (MRI) data (T1, diffusion, BOLD) acquired in 25 brain tumor patients before the tumor resection surgery, and six months after the surgery, together with the tumor masks, and in 11 controls (recruited among the patients’ caregivers). The dataset also contains behavioral and emotional scores obtained with standardized questionnaires. To simulate personalized computational models of the brain, we also provide structural connectivity matrices, necessary to perform whole-brain modelling with tools such as The Virtual Brain. In addition, we provide blood-oxygen-level-dependent imaging time series averaged across regions of interest for comparison with simulation results. An average resting state hemodynamic response function for each region of interest, as well as shape maps for each voxel, are also contributed.

## Abstract (summary)

**Table:** 

Measurement(s)	BOLD signal • Diffusion Anisotropy
Technology Type(s)	Functional Magnetic Resonance Imaging • Diffusion Weighted Imaging
Factor Type(s)	Surgery
Sample Characteristic - Organism	Homo sapiens

## Background & Summary

Noninvasive neuroimaging techniques such as functional MRI (fMRI) and diffusion-weighted imaging (DWI) fiber tracking are valuable tools to inform the presurgical process in the treatment of brain tumors^1^. An advantage of using whole brain imaging is that it allows to investigate the large-scale effect of surgery and the reorganization of the brain^2–4^. With the advent of large-scale generative modelling^5^, and the advances in noninvasive imaging of brain structure and function, new pieces can be added to the puzzle: mapping brain function and pathology to parameters of subject-specific computational models, and the possibility of performing virtual medical interventions and explore their consequences in the model.

The Virtual Brain (TVB)^6^ has established itself as a versatile and accessible neuroinformatics platform. In the last years its reach and accessibility has increased with its inclusion in the Human Brain Project (HBP), and then in EBRAINS, a platform for neuroscience data sharing and modelling, which is being developed by the HBP. EBRAINS provides many services for brain research and allows running analyses and simulations in the cloud^7^. Among the services are biologically inspired computational models at several spatial and temporal scales, providing the possibility of bridging them, as well as data, properly curated and organized according to the standards adopted by the community.

In TVB the activity of neural populations is simulated. This activity is then used to predict neuroimaging signals by using an appropriate transfer function. For BOLD data, the Balloon-Windkessel model^8^, or, more generally, a Hemodynamic Response Function (HRF), is used to simulate the coupling between neuronal activity and hemodynamics. While this model is robust and well-established, there is evidence that HRFs are region- and subject-specific^9^. Furthermore, differences between HRFs can confound the estimate of functional connectivity^10^. This could be even more relevant given that conditions, such as a brain tumor, or surgery, modulate the HRF. With this in mind, here we provide estimates of resting state HRFs, obtained with the rsHRF toolbox^11^, which can be used to obtain more personalized brain models.

The data presented here provide the basis for investigation on topics such as individual specificity of biophysical model parameters, differences in local model parameters dependent on distance from a tumor, and associations between model parameters and structural network topology and cognitive performance.

These data have been used for brain simulations of tumor patients before and after surgery^12,13^. The emotional scores have been analyzed separately^14^.

**Fig. 1:**
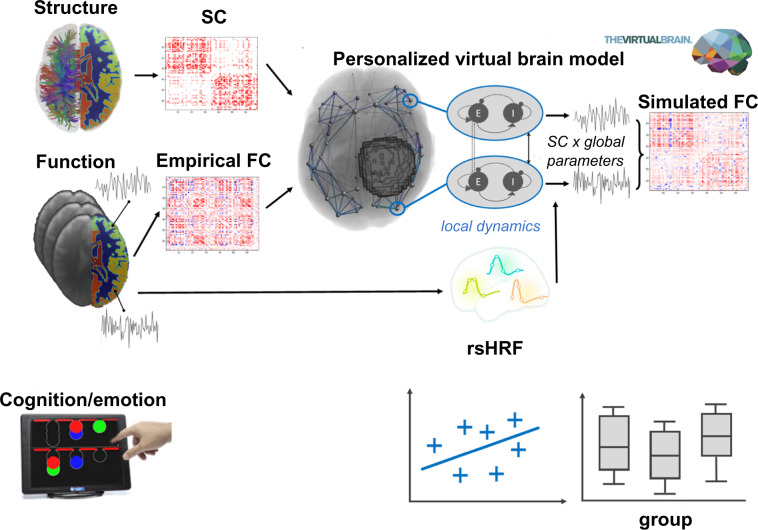
Data collection and analysis summary. Neuroimaging and behavioral data are acquired the day before the surgery, and approximately six months after the surgery. Empirical structural connectivity (SC) is used as input for the computational model. Simulated functional connectivity (FC) is compared with empirical FC. Subject- and region- specific hemodynamic response functions, retrieved via the rsHRF toolbox, are convolved with the simulated neural activity. A similar version of this figure appears in a previous study^12^ (copyright the authors).

Figure 1 reports a summary of the data and the analysis/modelling flowchart.

## Methods

The parts of this section referring to recruitment of subjects and data processing are necessarily quasi-identical versions of the descriptions in our related work^12^.

We included patients who were diagnosed with either a glioma, developing from glial cells, or a meningioma, developing in the meninges. Both types of tumors can be described by their malignancy, based on the World Health Organization (WHO) grading system. According to this system, grade I tumors are least malignant, whereas grade III (for meningioma) or IV (for glioma) tumors are most malignant. Malignancy relates to the speed with which the disease evolves, the extent to which the tumor infiltrates healthy brain tissue, and chances of recurrence or progression to higher grades of malignancy.

Patients were recruited at Ghent University Hospital (Belgium) between May 2015 and October 2017. Patients were eligible if they (1) were at least 18 years old, (2) had a supratentorial meningioma (WHO grade I or II) or glioma (WHO grade II or III) brain tumor, (3) were able to complete neuropsychological testing, and (4) were medically approved to undergo MRI investigation. Partners were also asked to participate in the study to constitute a group of control subjects that suffer from emotional distress comparable to that of the patients.

Participants were recruited on the day before patients’ surgery. All participants received detailed study information and gave written informed consent before study enrollment. This research was approved by the Ethics Committee at Ghent University Hospital.

We collected data from 11 glioma patients (mean age 47.5 y, SD = 11.3; 4 females), 14 meningioma patients (mean age 60.4 y, SD = 12.3; 11 females), and 11 healthy caregivers (mean age 58.6 y, SD = 10.3; 4 females).

From all participants, three types of MRI scans were obtained using a Siemens 3 T Magnetom Trio MRI scanner with a 32-channel head coil. First, T1-MPRAGE anatomic images were acquired (160 slices, TR = 1750 ms, TE = 4.18 ms, field of view = 256 mm, flip angle = 9°, voxel size = 1 × 1 × 1 mm, TA = 4:05 min). Next, resting-state functional echo-planar imaging (EPI) data were obtained in an interleaved order (42 slices, TR = 2100 ms, TE = 27 ms, field of view = 192 mm, flip angle = 90°, voxel size = 3 × 3 × 3 mm, TA = 6:24 min). After the first 4 control subjects, 5 meningioma patients, and 2 glioma patients were scanned, the fMRI protocol was accidentally changed to a TR of 2400 ms, resulting in a TA of 7:19 min. This has been taken care of in subsequent analyses by inclusion of an additional regressor in the model. During the fMRI scan, participants were instructed to keep their eyes closed and not fall asleep. Finally, a multishell high-angular resolution diffusion-weighted MRI (DWI) scan was acquired (60 slices; TR = 8700 ms; TE = 110 ms; field of view = 240 mm; 101 diffusion directions; b-values = 0, 700, 1200, 2800 s/mm2; voxel size = 2.5 × 2.5 × 2.5 mm; TA = 15:14 min). In addition, two DWI b = 0 s/mm2 images were collected with reversed phase-encoding blips for the purpose of correcting susceptibility-induced distortions.

In the first step, high-resolution anatomic images were processed using FreeSurfer to obtain a subject-specific parcellation of each subject’s brain into 68 cortical regions of interest (ROIs) (34 per hemisphere). T1-weighted data of all control subjects were subjected to the default recon-all processing pipeline, which includes the following steps: intensity normalization, skull stripping, removal of non-brain tissue, brain mask generation, cortical reconstruction, segmentation of subcortical white matter and deep gray matter volumetric structures, cortical tessellation of the gray matter/white matter and gray matter/pial boundary, and construction of a probabilistic atlas based cortical parcellation into 68 ROIs according to gyral and sulcal structures^15,16^.

As meningioma tumors generally exert pressure on the brain without infiltrating, our aim was to segment out the meningioma tumor before cortical reconstruction. However, visual inspection of the results showed this was done automatically by the recon-all processing pipeline of FreeSurfer in all but two meningioma patients. In the remaining two meningioma patients, who had very large lesions, manual edits were made.

Glioma tumors, in contrast, generally do infiltrate the brain. To obtain a whole-brain parcellation scheme for these patients, two additional steps were conducted. First, glioma tumors were segmented using the Unified Segmentation with Lesion toolbox^17^. Second, the Normalisation tool of the BCBtoolkit^18^ was used to produce an enantiomorphic filling of the affected area by symmetrically filling up the lesion mask with healthy tissue of the contralateral hemisphere^19^. These normalized anatomic MRI data were then processed using the standard recon-all FreeSurfer processing pipeline. Resulting parcellations were visually inspected and manually corrected in two glioma patients.

In glioma patients, tumor regions were defined as those cortical areas of the individual FreeSurfer parcellation that showed at least partial (i.e., minimum 1 voxel) overlap with the tumor mask. In meningioma patients, tumor regions consisted of regions that were (at least partially) displaced because of the tumor’s mass effect. To estimate which regions were displaced by the meningioma, patients’ anatomic images were transformed to MNI space (using FSL FLIRT with 12 DOF), and this transformation was applied to their tumor mask. Then, the overlap between subjects’ tumor mask in MNI space and the fsaverage Desikan–Killiany atlas in MNI space was calculated. Parcels that showed at least 1 voxel overlap with the tumor mask were denoted tumor nodes. Figure 3 displays the tumor masks overlapped for all the patients, in MNI space. This mask was generated with MRIcroGL^20^, available at https://www.nitrc.org/projects/mricrogl.

### Functional MRI preprocessing

fMRI data processing was conducted using FEAT (FMRI Expert Analysis Tool, version 6.00), part of FSL (FMRIB’s Software Library). Specifically, the following operations were applied: motion correction using MCFLIRT^21^, slice-timing correction, non-brain removal using BET^22^, grand-mean intensity normalization of the entire 4D dataset by a single multiplicative factor, and high-pass temporal filtering (100 s high-pass filter). Next, the FreeSurfer cortical parcellation obtained in the previous step was mapped to the subject’s functional space. To this end, fMRI images were linearly registered to the subject’s high-resolution T1-weighted images using the epi_reg function of FSL FLIRT^21,23^, after which the inverse of this transformation matrix was applied to transform the FreeSurfer parcellation scheme to the subject’s functional space. Average BOLD signal time series for each region were then generated by computing the spatial mean for all voxel time-series of each region. Lastly, functional connectivity (FC) matrices were constructed by calculating the Fisher *z*-transformed Pearson correlation coefficient between all pairs of region-wise aggregated BOLD time series.

### Diffusion-weighted MRI preprocessing

Because all analyses of this study depend on the quality of the structural connectivity matrices, a state-of-the art pipeline was constructed for the preprocessing of DWI data and consecutive network construction, using a combination of FSL (version 5.0.9) and MRtrix3 (http://www.mrtrix.org; version 0.3.RC2). First, raw diffusion-weighted MRI images were corrected for several artifacts. In particular, DWI images were denoised (MRtrix dwidenoise^24^) and corrected for Gibbs ringing artifacts (MRtrix mrdegibbs^25^), for motion and eddy currents (FSL eddy^26^), for susceptibility-induced distortions (FSL topup^27^), and for bias field inhomogeneities (FSL FAST^28^). Next, subjects’ high-resolution anatomic images were linearly registered to diffusion space with the epi_reg function of FSL FLIRT^21,23^ and segmented into gray matter, white matter, and cerebrospinal fluid (FSL FAST^28^).

In glioma patients, tumor regions were defined as those cortical areas of the individual FreeSurfer parcellation that showed at least partial (i.e., minimum 1 voxel) overlap with the tumor mask. In meningioma patients, tumor regions consisted of regions that were (at least partially) displaced because of the tumor’s mass effect. To estimate which regions were displaced by the meningioma, patients’ anatomic images were transformed to MNI space (using FSL FLIRT with 12 DOF), and this transformation was applied to their tumor mask. Then, the overlap between subjects’ tumor mask in MNI space and the fsaverage Desikan–Killiani atlas^16^ in MNI space was calculated. Parcels that showed at least 1 voxel overlap with the tumor mask were denoted tumor nodes.

DWI images were then intensity-normalized across subjects, and group average response functions were calculated. Specifically, response functions for each subject were estimated per b-value shell (b = 0, 700, 1200, and 2800 s/mm^2^) and per tissue type (white matter, gray matter, cerebrospinal fluid) using the MRtrix3 script dwi2response dhollander^29^. A scaling factor per subject was calculated by which the individual response functions could be multiplied to obtain the average response function across all subjects. DWI images were then initially normalized by dividing subjects’ DWI images by their corresponding scaling factor. After that, response functions per b-value shell and tissue type were recalculated for every subject and averaged across all subjects. This set of group average response functions was subsequently used in multi-shell multi-tissue constrained spherical deconvolution to estimate the fiber orientation distributions (MRtrix3 msdwi2fod^30^). In addition, tissue components from multi-tissue CSD were once more intensity normalized using MRtrix3 mtnormalise.

Next, anatomically constrained probabilistic whole-brain fiber tracking (ACT) was performed using dynamic seeding generating 30 million streamlines per subject (MRtrix3 tckgen^31,32^). Afterward, spherical-deconvolution informed filtering of tractograms (SIFT) was applied to selectively filter out streamlines from the tractogram to improve the fit between the streamline reconstruction and the underlying diffusion images, retaining 7.5 million streamlines per subject (MRtrix3 sift^31^). An SC matrix was then constructed by transforming the individual’s FreeSurfer parcellation scheme to diffusion space and calculating the number of estimated tracts between any two brain regions (MRtrix3 tck2connectome). In addition, a distance matrix was constructed by calculating the average length of all streamlines connecting any two nodes (MRtrix3 tck2connectome). By using a proper high-order model and taking into account the full fiber orientation distribution function (through CSD), by taking into account the presence of non–white matter tissue (through multi-tissue CSD), by applying realistic individual anatomic priors (through ACT), and by ensuring fidelity of the tractograms to the data (through SIFT), it has been shown that the biological accuracy of tractograms can be vastly increased compared with those obtained with unfiltered unconstrained diffusion tensor tracking^33^.

**Fig. 2:**
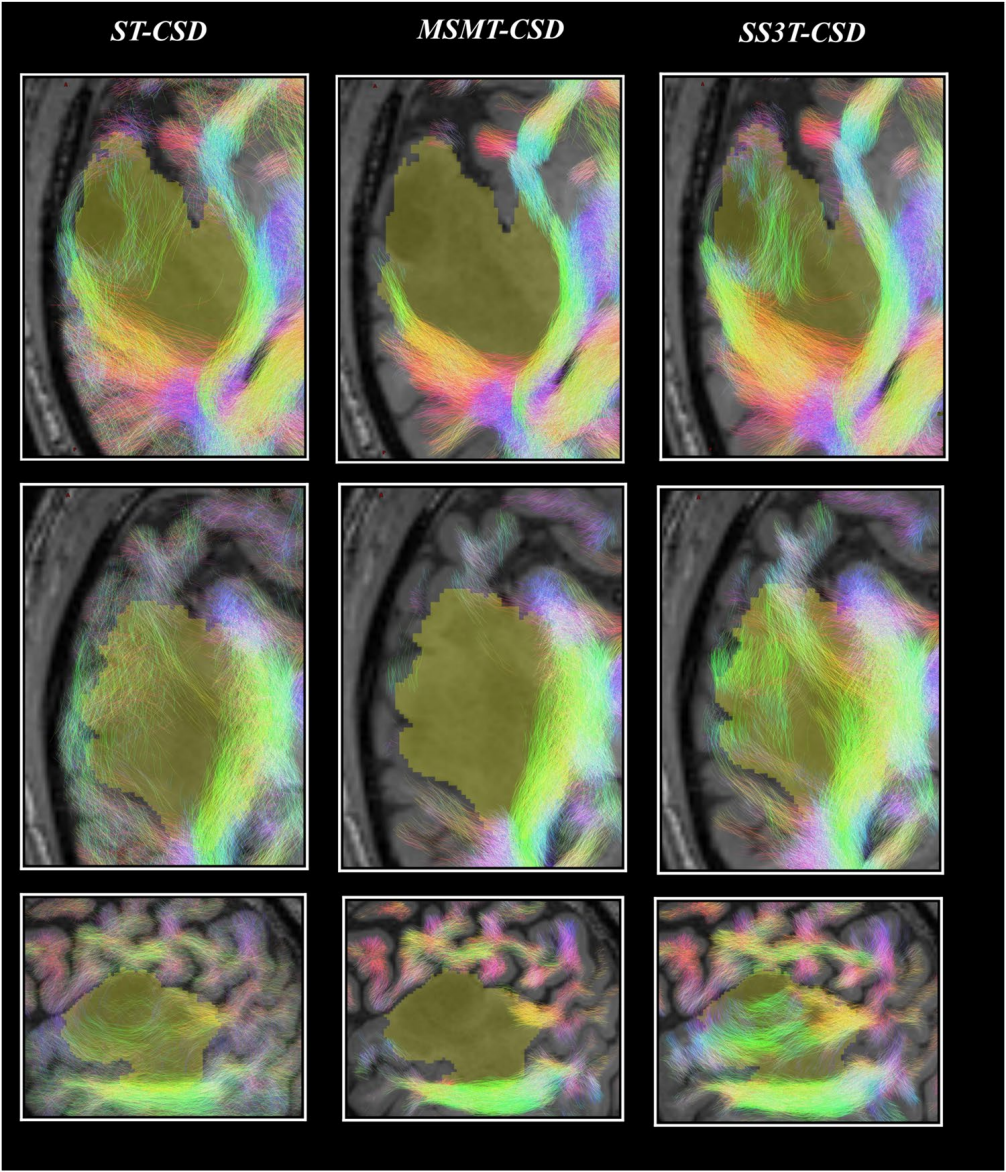
Comparison of tractography results based on single tissue (ST)-CSD, Multi Shell Multi Tissue (MSMT)-CSD and Single Shell 3 Tissues (SS3T-CSD)^34^ pipelines for patient PAT26 (anaplastic astrocytoma WHO grade III). Each result is overlaid on the T1-weighted image and the tumor segmentation is shown in yellow (at the spatial resolution of the dMRI data). Streamlines are colored using the DEC convention (red: left-right; green: anterior-posterior; blue: superior-inferior) and shown within a 2.5 mm thick “slab” centered around the slice. Each row shows a different slice. Top row: axial slice. Middle row: other axial slice, further down the tumor volume. Bottom row: sagittal slice through the tumor volume. Adapted from a previous study^34^, copyright the authors.

Particular care was taken in assuring the maximum of reliability of diffusion in the peri-tumoral regions using 3-tissue constrained spherical deconvolution^34^. Figure 2 reports a comparison of Single-Tissue, Multi Shell Multi Tissue, and Single Shell 3 Tissues CSD. Other examples are found in the dedicated study^34^. For both 3-tissue CSD techniques (MSMT-CSD and SS3T-CSD), we expected WM FODs to be smaller in the tumor regions, reflecting a reduced presence of healthy axons due to infiltration of tumor tissue (as diffusion signal resulting from the tumor tissue might be “picked up” by the non-WM compartments in the model instead) and other potential sources of WM damage. To address this challenge, we devised a pragmatic solution where we gradually reduced the FOD amplitude threshold close to and even more so within the tumor. To this end, we first registered the T1-weighted image to the dMRI data using FSL’s registration tools (FLIRT) (Jenkinson *et al*., 2002; Jenkinson and Smith, 2001). Next, tumors were manually delineated based on the T1-weighted images, and further automatically optimized using the Unified Segmentation with Lesion toolbox (Phillips and Pernet, 2017). These tumor segmentations were then spatially smoothed using a Gaussian kernel with a standard deviation of 3 mm, to introduce a smooth boundary extending slightly beyond—as well as within—the edges of the tumor. Finally, during the actual tractography process, the FOD amplitude threshold was reduced by up to a factor 3 within the tumor, modulated by the smoothed tumor segmentation Fig. 2

**Fig. 3:**
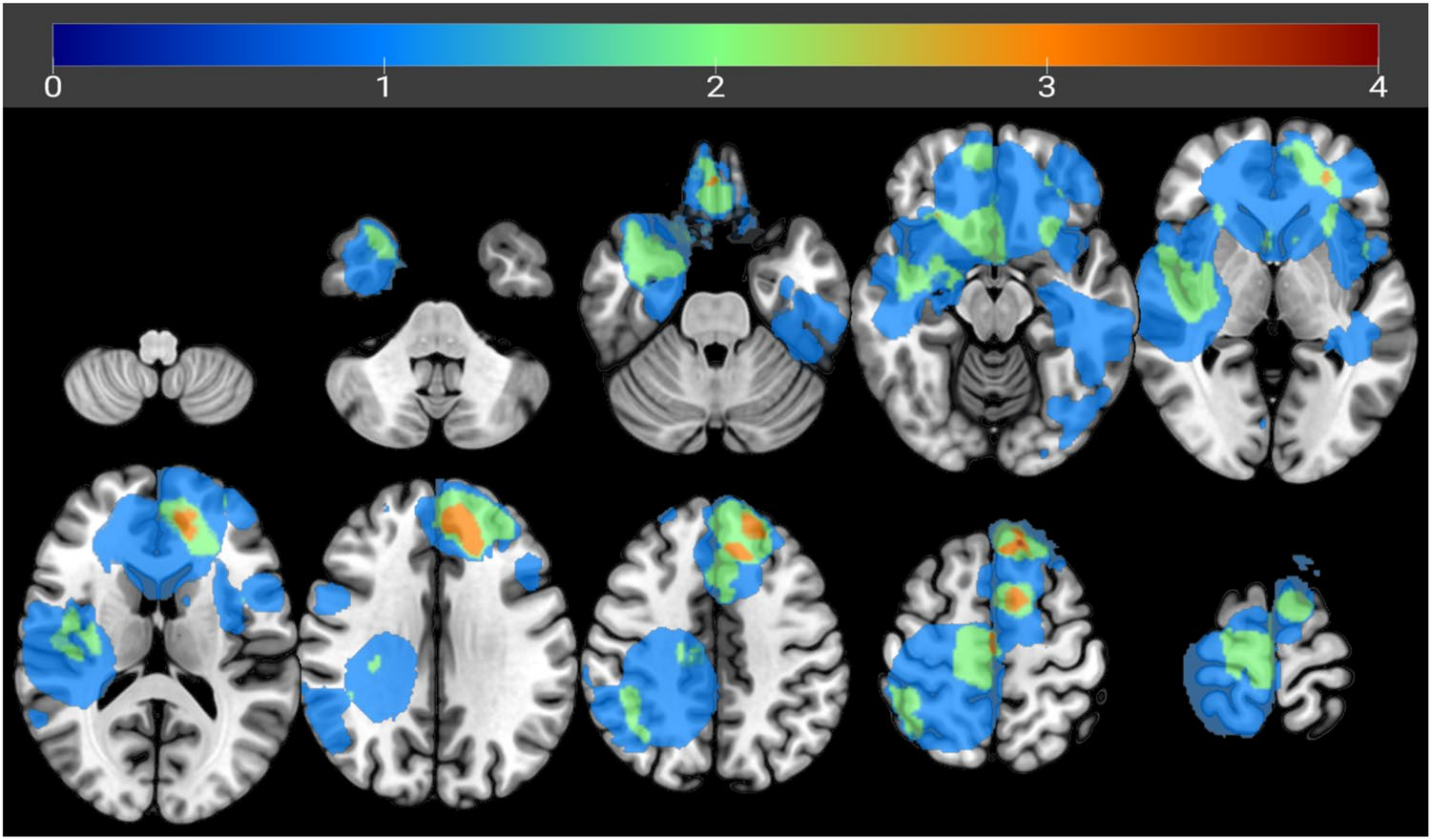
Overlay of the tumor masks. The tumor masks in MNI space are overlaid. The color indicates the number of subjects with a tumor at the location.

### Behavioral data

The Emotion Regulation Questionnaire (ERQ)^35^ was used to measure cognitive reappraisal and expressive suppression. Cognitive performance was assessed using the Cambridge Neuropsychological Test Automated Battery (CANTAB; Cambridge Cognition (2017); All rights reserved; http://www.cambridgecognition.com). In particular, four cognitive domains were examined that have been identified by previous studies to be frequently affected by brain tumors: sustained attention (Rapid Visual Information Processing [RVP]), working memory (Spatial Span [SSP]), information processing speed (Reaction Time [RTI]), and executive functioning (Stockings of Cambridge [SOC])^36^. All tests were administered in random order to avoid sequence bias. To measure the extent to which people generally tend to worry, the Penn State Worry Questionnaire (PSWQ)^37^ was used. A more extended description of these measures and their analyses is reported in a dedicated study^14^. Handedness was calculated by means of the Edinburgh Handedness Inventory^38^.

Lastly, we thresholded and normalized the resulting SC matrices. Thresholding was conducted to minimize false-positive streamlines. Using an absolute threshold (setting to zero all connection weights smaller than 5) yielded a decaying degree distribution as those observed in invasive anatomical studies yet ensuring that all subjects’ network remained fully connected. Normalization was performed by dividing all SC weights by a constant scalar across subjects (75,000 in our case: 7.5 million streamlines generated per subject/100) to ensure all SC weights varied between 0 and 1, which was required for computational modeling in TVB.

## Data Records

The basic clinical and demographic details of the subjects are in Table 1 in the first study presenting this dataset^12^.

The MRI, fMRI, and DWI data are stored on OpenNeuro.org, with records https://openneuro.org/datasets/ds001226^39^ and https://openneuro.org/datasets/ds002080^40^ for the pre-operatory and post-operatory dataset, respectively.

The “derivatives” folder contains five subfolders. The “tumor masks” for the patients, where brain regions interested by the tumor were delineated in a semiautomated and manual way by the lead researcher (HA). The masks are reported both in the subject’s native space (*T1) and in a normalized MNI neurological space (*MNI). The _DM suffix stands for “Disconnection Mask”.

The”mriqc” derivative folders contain the results of the functional and anatomical image quality control as described in the “Technical Validation” section.

The”dmriqc” derivative folders contain the results of the diffusion image quality control as described in the “Technical Validation” section.

The”rsHRF” derivative folders contain the maps of three shape parameters (height, time to peak, full width at half maximum) for the resting state hemodynamic response function obtained applying the rsHRF toolbox^11^.

The”TVB” derivatives folders contain everything needed to perform personalized large-scale computational modelling using TVB, according to the following structure:

**derivatives/**

- **TVB/**
  - dataset_description.json: details of the dataset as required by BIDS
  - CHANGE: log of changes since first submission
  - README: basic info on the dataset
  - parcellation.txt: description of the ROIs, names and locations
  - participants.tsv: information on the subjects participating in this experiment.
  - **sub-XXX/**: folder of the subjects (PAT for patients, CON for controls)
    - **ses-XXX/**: session (preop/postop)
      - **SC.zip:** zip file with structural connectivity, as in
        - Centres.txt
        - 1st columns → ROI name
        - 2nd to 4th column → 3D coordinates
        - Weights.txt → n*n matrix, where n is the number of regions in the SC
        - Tract_lengths.txt → n*n matrix, containing region distances
        - Areas.txt → column with space [mm^2] of the cortical areas
        - Cortical.txt → binary column with 1 if region is cortical and 0 if region is subcortical
        - Average_orientations.txt → 3D coordinates for orientation of the normal vector in each region
      - **SCthrAn.mat**: thresholded structural connectivity, as described in^12^
      - **FC.mat**: matrix of Pearson correlation coefficients between processed time series extracted from the ROIs
      - **ROIts.dat** time series of the BOLD signal averaged over the voxels in each ROI, after preprocessing
      - **HRF.mat** shape parameters (baseline, height, time to peak, full width at half maximum, of the resting state hemodynamic response function), for each of the 68 ROIs
      - **HRF.csv** time courses of the HRF for each of the 68 ROIs, upsampled to the synaptic time scales used for modelling neural activity in TVB (0.1 msec).

These data are also hosted on EBRAINS^41^. Upon registration, users can perform computational analyses on the cloud, using the dedicated notebooks.

The behavioral data and the results of the cognitive tests are stored on the Open Science Framework^42^.

## Technical Validation

An initial review of the data quality is provided using a variety of image quality metrics derived with MRIQC v21.0.0rc2^43^. MRIQC was run using Singularity and the singularity image was built from its docker hub container (https://hub.docker.com/r/nipreps/mriqc/). The outputs include a variety of image quality metrics per scan, that is also summarized across the entire dataset. Note, that here we did not exclude any subjects based on the resulting quality metrics, and the resulting scores could be useful for future researchers aiming to filter scans according to quality.

**Fig. 4:**
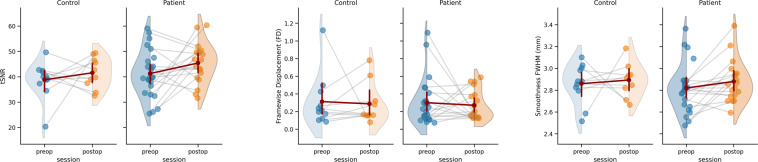
Summary of image quality metrics in terms of temporal signal-to-noise ratio (tSNR), Framewise Displacement, and Spatial Smoothness.

In Fig. 4 we briefly summarize the results of three common metrics that capture head motion, spatial smoothness and temporal signal quality for the functional data. The temporal-signal-to-noise ratio (tSNR) was computed as a measure of the signal quality using MRIQC. The mean tSNR across all subjects and sessions was 42.119 (SD = 8.154). Head motion is summarized by deriving the mean framewise displacement (FD) for each functional scan across all subjects and sessions. The median FD across all sessions and subjects was 0.201 mm (SD = 0.231 mm). Spatial smoothness was quantified based on the raw functional data using the 3dFWHMx function in AFN 22.0.5I. Smoothness was estimated from each subject’s native brain-space within a predefined brain mask. In addition, time series were temporally detrended before the smoothness was estimated.

These metrics fall within the range of the datasets considered in the MRIQC collection, as well as comparable with other clinical data collected before brain surgery^44^. Some high values of framewise displacement (FD > 0.5) are present. While these values are expected in elderly neurological patients, researchers might choose to censor frames with high displacement.

**Fig. 5:**
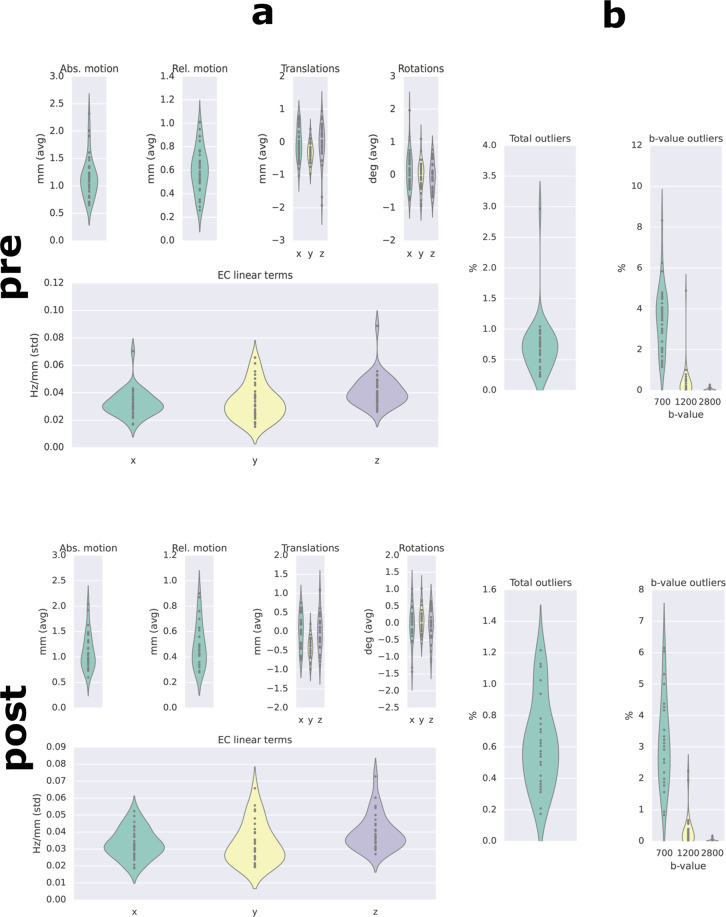
Image quality metrics (IQMs) for the DWI data. Upper panels show IQMs for pre-surgery DWI data, lower panels show IQMs for post-surgery DWI data. Panel A shows the average absolute motion, average relative motion (i.e., with respect to the previous volume), as well as average translations and rotations in three dimensions.This panel also shows the standard deviation of the linear terms of the eddy current-induced field for each subject. Panel B shows the total percentage of slices labeled as outliers^46^ by FSL’s eddy for each subject (left graphs) and the percentage of outliers as a function of the b-value (right graphs). Control subject six showed a slightly higher percentage of outliers than the other subjects, but still fell within acceptable ranges (<3.0%). Dots represent individual subjects, width of the violin plots reflects the density of subjects.

The quality of the DWI data was assessed using FSL’s eddyqc^45^ tool (see, https://fsl.fmrib.ox.ac.uk/fsl/fslwiki/eddyqc). Figure 5 gives an overview of the image quality metrics (IQMs) in our sample. We followed a procedure for Incorporating outlier detection and replacement into a non-parametric framework for movement and distortion correction of diffusion MR images^46^. All metrics fell within acceptable ranges. Extensive subject-level quality reports including additional quality metrics produced using dmriqc_flow^47^ can be found in the *derivatives* folder on openneuro.org for the two datasets.

The shape of the retrieved HRF was validated using rodent data and simulations, as described in the rsHRF toolbox paper^11^.

The validation of the pipeline used to prepare the data for computational modelling with TVB is validated in line with the field standards as described in the accompanying paper^48^.

## Usage Notes

Case studies presenting the application of these data to TVB ecosystem are present on EBRAINS, and described in this educational video https://www.thevirtualbrain.org/tvb/zwei/newswire-educase/single/42279-learn-modeling-brain-dynamics-in-brain-tumor-patients-using-the-virtual-brain.

## Data Availability

Code for the TVB Brain Tumor pipeline is available at https://github.com/haerts/The-Virtual-Brain-Tumor-Patient. Code for retrieving the voxelwise resting state hemodynamic response function is available at https://github.com/bids-apps/rsHRF. Proof-of-concept notebooks for the introduction of region- and subject-specific HRF in TVB are presented at https://github.com/AmoghJohri/TVB-Tests. The TVB processing pipeline is available at https://github.com/BrainModes/TVB-empirical-data-pipeline and https://search.kg.ebrains.eu/instances/Software/71265c9f-5fe3-40e3-a7e4-b2bb45b5ea6e for cloud computing.
